# Evaluation of diagnostic time in pediatric patients with eosinophilic gastrointestinal disorders according to their clinical features

**DOI:** 10.1186/s13052-023-01410-1

**Published:** 2023-01-16

**Authors:** Martina Votto, Marco Vincenzo Lenti, Annalisa De Silvestri, Francesca Bertaina, Mirko Bertozzi, Silvia Caimmi, Emanuele Cereda, Maria De Filippo, Antonio Di Sabatino, Catherine Klersy, Alessandro Raffaele, Giovanna Riccipetitoni, Gian Luigi Marseglia, Amelia Licari, Ilaria Brambilla

**Affiliations:** 1 Department of Clinical, Surgical, Diagnostic and Pediatric Sciences, University of Pavia, Pavia, Italy; 2 First Department of Internal Medicine, San Matteo Hospital Foundation, University of Pavia, Pavia, Italy; 3 Scientific Direction, Clinical Epidemiology and Biometric Unit, Fondazione IRCCS Policlinico San Matteo, Pavia, Italy; 4 Pediatric Surgery Unit, Department of Maternal and Child Health, Fondazione IRCCS-Policlinico San Matteo, Pavia, Italy; 5 Pediatric Clinic, Fondazione IRCCS Policlinico San Matteo, Pavia, Italy; 6 Clinical Nutrition and Dietetics Unit, Fondazione IRCCS Policlinico San Matteo, 27100 Pavia, Italy

**Keywords:** Adolescents, Children, Diagnostic time, Eosinophilic esophagitis, Failure to thrive, Growth, Non-esophageal eosinophilic gastrointestinal disorders

## Abstract

Eosinophilic gastrointestinal disorders (EGIDs) are chronic/remittent inflammatory diseases associated with a substantial diagnostic delay, often attributable to misdiagnosis and variable clinical presentation in adults. In the pediatric population, few studies have been conducted worldwide reporting EGID diagnostic delay and its consequences on patients. This study aims to analyze and identify potential clinical factors and complications associated with a longer diagnostic time. We performed a retrospective analysis of pediatric patients with EGIDs followed at the Center for Pediatric EGIDs in Pavia, Italy. A total of 60 patients with EGIDs were enrolled. Thirty-nine (65%) patients had EoE, and 21 (35%) non-esophageal EGIDs. EGID diagnosis was achieved about 2 years after the symptom onset, and the median diagnostic time was 12 months (IQR 12–24 months). Diagnostic time was 12 months (IQR 12–69) in non-esophageal EGIDs and 12 months (IQR 4–24 months) in EoE patients. EoE patients presenting with FTT and feeding issues experienced a longer diagnostic time (*p* = 0.02 and *p* = 0.05, respectively) than children without growth and feeding impairments.

In this study, symptoms appeared about 2 years before the definitive EGID diagnosis was reached, and this diagnostic time was shorter than the delay observed in other published studies. Especially in EoE children, the diagnostic time is significantly associated with impaired child growth, highlighting the importance of an early diagnosis to prevent esophageal stenosis and failure to thrive.

To the Editor,

Eosinophilic gastrointestinal disorders (EGIDs) are clinically heterogeneous chronic diseases with non-specific symptoms that vary with age and the site of pathological eosinophilic gastrointestinal (GI) inflammation [1]. As a consequence, EGIDs are associated with a substantial diagnostic delay, often attributable to misdiagnosis and variable clinical presentation in adults [2]. Instead, in the pediatric population, only a few studies have been conducted worldwide reporting EGID diagnostic delay, its risk factors, and its consequences on patients [3, 4]. Therefore, this study aims to analyze the time from symptom onset to EGID diagnosis and identify potential clinical factors or predictable complications associated with a longer diagnostic delay.

We performed a retrospective analysis (from June 2021 to July 2022) of pediatric patients followed at the Center for Pediatric Eosinophilic GI Disorders (CPED) in Pavia, Italy. Patients enrolled were younger than 19 years at the time of the EGID diagnosis. EGIDs have been categorized into eosinophilic esophagitis (EoE) and non-esophageal EGIDs. Diagnosis of EoE was defined as ≥ 15 eosinophils/high power field identified in at least one esophageal biopsy [5]. There are no universal guidelines for the diagnosis of non-esophageal EGIDs; therefore, pathology reports were reviewed according to the cut-offs proposed by Collins et al. [6] All children with other causes of intestinal eosinophilic inflammation (i.e., inflammatory bowel diseases, parasite infections, intestinal vasculitis, malignancies) were excluded. Data collected from enrolled patients included demographics (date of birth, age at diagnosis and symptoms onset, gender, ethnicity), medical history of coexisting atopic diseases (allergic rhinitis, asthma, atopic dermatitis, and food allergy), and symptoms at the time of diagnosis. In EoE patients, endoscopic findings have been reported according to the validated EoE endoscopic reference score (EREFS) [7]. Diagnostic time was estimated as the time-lapse (months) between the onset of symptoms and the final diagnosis of EGIDs. All data were extracted from electronic medical records and semi-anonymized. The Ethical Committee approved this study (protocol number 0003241/22, GOLDEN study, NCT05219903). All patients provided written informed consent, according to the Declaration of Helsinki and more recent amendments [8, 9]. Continuous data were described with median and interquartile range (IQR; i.e., 25th–75th percentiles), whereas categorical data as counts and percentages. Comparative analysis was performed using the Mann Whitey U and Fisher exact tests. The Kruskal Wallis test was used to compare the diagnostic time through different age ranges (≤ 1 year, 1–5, 6–11, and ≥ 12 years). Statistical significance was set at p ≤ 0.05. The statistical analyses were performed through Stata v17 (StataCorp USA 2020).

A total of 60 patients with EGIDs were enrolled. Thirty-nine (65%) patients had EoE, and 21 (35%) non-esophageal EGIDs (Table 1). Most enrolled EGID patients were males (70%) and Caucasians (88%). Overall, 63% of the enrolled patients showed other coexisting allergic diseases that were more evident in the EoE patients (70%) compared to non-esophageal EGID forms (52%). Food impaction, dysphagia, and feeding issues were only reported in patients with EoE (21%, 23%, and 18%, respectively); on the other hand, diarrhea with weight loss specifically depicted the non-esophageal EGID forms (38% and 14%, respectively). Failure to thrive (FTT) was found in 20% of all EGID patients (26% of EoE and 10% of non-esophageal EGID patients, respectively). About 76% of all patients received an EGID diagnosis during school age and adolescence; in particular, EoE and non-esophageal EGIDs were diagnosed in 35.9 and 52.4% of patients 6–11 years old, respectively (Table 1). EGID symptoms appeared at a median age of 8 years (IQR 3–11 years). EGID diagnosis was achieved about 2 years after the symptom onset, and the median diagnostic time was 12 months (IQR 12–24 months) (Table 2). Diagnostic time was 12 months (IQR 12–69) in non-esophageal EGIDs and 12 months (IQR 4–24 months) in EoE patients. In the EGID cohort, the longest diagnostic time was registered among school-aged children (24 [IQR 8–54] months) and adolescents (12 [12–30] months) compared to other age ranges. No significant differences in diagnostic time were found according to sex and allergic comorbidities in EoE and non-esophageal EGID patients. EoE patients presenting with FTT and feeding issues experienced a longer diagnostic time (*p* = 0.02 and *p* = 0.05, respectively) than children without growth and feeding impairments (Fig. 1). Suggestive symptoms of EoE, such as food impaction and dysphagia, were not significantly associated with a shorter diagnostic time (*p* = 0.21 and *p* = 0.61, respectively). Similarly, the diagnostic time in non-esophageal EGID patients with FTT was longer than that found in children without FTT, although this difference was not statistically significant (*p* = 0.53) (Fig. 1). In non-esophageal EGID patients, neither diarrhea nor abdominal pain was related to a shorter diagnostic time (*p* = 0.92 and *p* = 0.82, respectively). In the EoE cohort, the finding of a fibro-stenotic phenotype (esophageal fixed rings and structures) was not associated with a longer diagnostic time compared to patients with an inflammatory endoscopic pattern (mucosal edema, furrows, white exudates).

**Table 1:** Demographic and clinical features of enrolled EGID patients

	Overall	EoE	Non-esophageal EGIDs
EGID patients, *n (%)*	60 (100)	39 (65)	21 (35)
Male, *n (%)*	42 (70)	29 (74)	14 (67)
Caucasian, *n (%)*	53 (88)	33 (85)	20 (95)
Age at diagnosis			
≤ 1 year, *n (%)*	4 (6.7)	3 (7.8)	1 (4.8)
1–5 years, *n (%)*	10 (16.7)	9 (23)	1 (4.8)
6–11 years, *n (%)*	25 (41.6)	14 (35.9)	11 (52.4)
≥ 12 years, *n (%)*	21 (35)	13 (33.3)	8 (38.0)
Coexisting allergic diseases, *n (%)*	38 (63)	27 (70)	11 (52)
Allergic rhinitis, *n (%)*	30 (50)	19 (49)	11 (52)
Asthma, *n (%)*	12 (20)	10 (26)	2 (10)
Atopic dermatitis, *n (%)*	11 (18)	9 (23)	2 (10)
Food allergy, *n (%)*	15 (25)	13 (33)	2 (10)
Symptoms			
Abdominal pain, *n (%)*	32 (53)	14 (36)	18 (86)
Diarrhea, *n (%)*	8 (13)	0 (0)	8 (38)
Dysphagia, *n (%)*	9 (23)	9 (23)	0 (0)
Failure to thrive, *n (%)*	12 (20)	10 (26)	2 (10)
Food impaction, *n (%)*	8 (13)	8 (21)	0 (0)
GERD-like symptoms, *n (%)*	20 (33)	20 (51)	0 (0)
Nausea and vomiting, *n (%)*	15 (25)	12 (31)	3 (14)
Reduced appetite and feeding issues, *n (%)*	7 (12)	7 (18)	0 (0)
Weight loss, *n (%)*	3 (5)	0 (0)	3 (14)
Endoscopic findings			
Edema, *n (%)*	19 (32)	19 (49)	0 (0)
Rings, *n (%)*	9 (15)	9 (23)	0 (0)
Exudates, *n (%)*	6 (10)	6 (15)	0 (0)
Furrows, *n (%)*	7 (12)	7 (18)	0 (0)
Stricture, *n (%)*	2 (3)	2 (5)	0 (0)
Normal mucosa, *n (%)*	11 (18)	0 (0)	11 (52)
Nodular lymphoid hyperplasia, *n (%)*	5 (8)	0 (0)	5 (24)
Mucosal inflammation, *n (%)*	5 (8)	0 (0)	5 (24)

*EGIDs* eosinophilic gastrointestinal disorders, *EoE* eosinophilic esophagitis

**Table 2:** Diagnostic time according to clinical features of EGID patients

**Overall EGID patients**	**Diagnostic time (months)** ***median (IQR)***^a^	***p*-value**
**Age at symptoms onset**, *years*	8 (3 – 11)	-
≤ 1 year	4.5 (1.5 – 10)	***0.04*** (1 year vs. 12 years)
1 – 5 years	12 (1 – 15)	
6 – 11 years	24 (8 – 54)	
≥ 12 years	12 (12 – 30)	
**Age at diagnosis,***years*	10 (6 – 13)	-
**Diagnostic time**, *months*	12 (12 –24)	-
**Eosinophilic esophagitis**	**Diagnostic time (months)** ***median (IQR)***^a^	***p*-value**
**Sex**		
Male	12 (4.5 – 24)	0.57
Female	12 (3 – 22)	
**Age at symptoms onset**,* years*	8 (3 - 12)	-
≤ 1 year	3 (1 – 6)	n.s.
1 – 5 years	12 (1 – 17.5)	
6 – 11 years	12 (3.3 – 30)	
≥ 12 years	12 (12 – 30)	
**Age at diagnosis**,* years*	10 (4 – 14)	-
**Diagnostic time***, months*	12 (4 – 24)	-
**Comorbidities**		
Allergic diseases		
Yes	12 (4 – 24)	0.97
No	17 (1.5 – 26)	
**Symptoms**		
Dysphagia		
Yes	12 (8 – 30)	0.61
No	12 (3 – 24)	
Food impaction		
Yes	18 (12 – 33)	0.21
No	12 (3 – 23)	
Feeding issues and reduced appetite		
Yes	24 (12 – 27)	0.05
No	12 (3 – 23)	
Failure to thrive		
Yes	25.5 (12 – 48)	***0.02***
No	12 (3.5 – 23.5)	
GERD-like symptoms		
Yes	12 (4.5 – 24)	0.80
No	12 (3 – 24)	
Nausea and vomiting		
Yes	8 (1 – 21)	0.10
No	12 (12 – 24)	
Abdominal pain		
Yes	12 (4 – 39)	0.42
No	12 (2 – 23)	
**Endoscopic pattern **		
Fibro-stenotic pattern^b^		
Yes	12 (4 – 27)	0.90
No	12 (4 – 24)	
**Non-esophageal EGIDs**	**Diagnostic time (months)** ***median (IQR)***^a^	***p*-value**
**Sex**		
Male	18 (12 – 82.5)	0.31
Female	12 (11 – 60)	
**Age at symptoms onset**,* years*	7 (3 – 11)	-
≤ 1 year	11 (11 – 11)	n.s.
1 – 5 years	12 (12 – 12)	
6 – 11 years	24 (12 – 78)	
≥ 12 years	12 (12 – 69)	
**Age at diagnosis**,* years*	11 (6.5 – 12)	-
**Diagnostic time***, months*	12 (12 – 69)	-
**Comorbidities**		
Allergic diseases		
Yes	18 (12 – 69)	0.87
No	12 (12 – 69)	
**Symptoms**		
Failure to thrive		
Yes	90 (12 – 168)	0.53
No	12 (12 – 60)	
Weight loss		
Yes	12 (11 – 96)	0.67
No	18 (12 – 64.5)	
Nausea and vomiting		
Yes	24 (12 – 60)	0.82
No	12 (12 – 79.5)	
Abdominal pain		
Yes	12 (0 – 79.5)	0.82
No	24 (12 – 60)	
Diarrhea		
Yes	18 (12 – 51)	0.92
No	12 (12 – 90)	

*EGIDs* eosinophilic gastrointestinal disorders, *EoE* eosinophilic esophagitis, *IQR* interquartile range, *SD* standard deviation

^a^25^th^-75^th^ percentiles

^b^Esophageal rings and stricture

**Fig. 1:**
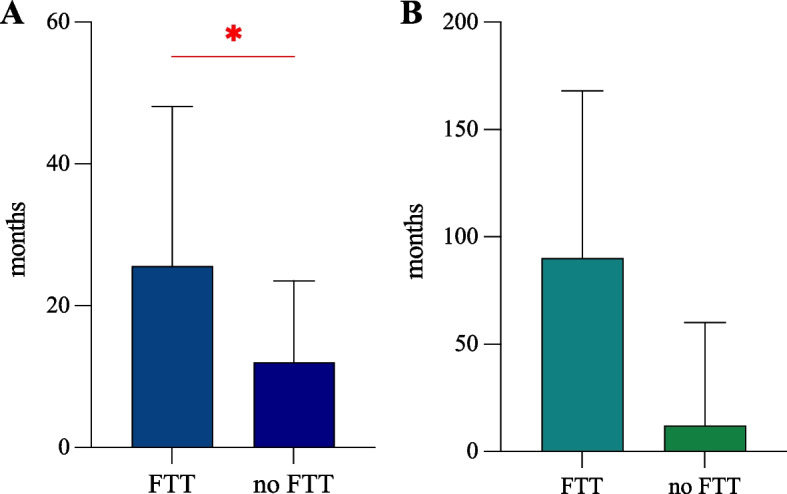
Box plots displaying the median and interquartile range of diagnostic time in EoE **(A)** and non-esophageal EGID patients **(B)**. Diagnostic time (months, y-axis) is higher in EGID children and adolescents with failure to thrive (FTT, x-axis) compared to those without (*p* = 0.02 and *p* = 0.53 in EoE [A] and non-esophageal EGIDs [B], respectively)

In this study, symptoms appeared about 2 years before the definitive EGID diagnosis was reached, and this diagnostic time was shorter than the delay observed in other published studies. Nevertheless, studies assessing the diagnostic delay in EGID patients are limited and have been mainly realized in adults [2–4]. Schoepfer et al. observed a median diagnostic delay of 6 years that was longer in the first two decades of life [3]. Conversely, a registry of 705 EoE patients highlighted that the diagnostic delay was higher in adults than in pediatric patients [10]. Only one study assessed the diagnostic delay in non-esophageal EGID patients, reporting a mean delay of 3.6 years that was longer in adults than children [4]. Lenti et al. identified an overall diagnostic delay of 36 months and found at least one previous misdiagnosis in 41.8% of adults with EoE [2]. Similarly, Chehade et al. found that 44.3% of patients with eosinophilic gastritis/duodenitis received a documented diagnosis of another gastrointestinal condition before the definitive diagnosis [4]. These data, together with the finding of a shorter diagnostic time in infancy in our cohort, suggest that toddlers and young children are less likely to receive an alternative diagnosis and the spectrum of differential diagnoses for pediatric patients is not as broad as for adult patients or adolescents. Initially, all enrolled patients, especially adolescents, were treated as functional GI disorders or gastroesophageal reflux disease, prolonging the diagnostic time; however, the non-response to conservative treatments allowed us to perform GI endoscopy. Esophageal strictures generally correlate with the duration of untreated disease and a longer diagnostic delay period [3]. In the Swiss study, Schoepfer et al. found that the diagnostic delay was the only risk factor for esophageal stenosis at the time of EoE diagnosis [3]. This correlation was not confirmed by our results; however, this discrepancy may be explained by the small pediatric population enrolled and a shorter diagnostic time than that reported in the Swiss study. Finally, the shorter diagnostic time observed in our cohort may be further related to the fact that patients are followed in a third-level Hospital with a multidisciplinary pediatric team and specialized pediatric endoscopists.

Notably, we observed a high diagnostic time in children with non-esophageal EGIDs whose symptoms are heterogeneous, non-specific, and often misdiagnosed with other more common GI disorders, such as functional GI disorders [1]. The clinical heterogeneity of EGIDs and the absence of specific non-invasive biomarkers are probably the main limitations to a prompt diagnosis and a shorter diagnostic process, especially in non-esophageal EGID cases.

This study first identified that the diagnostic time is significantly associated with impaired child growth in children with EGIDs, probably due to the prolonged intestinal inflammation (that worsens feeding issues and nutritional status) and more differential diagnoses of FTT in comparison to other more suggestive GI symptoms like dysphagia or food impaction [11]. Common GI inflammatory disorders, such as celiac disease, are often associated with FTT, weight loss, and delayed puberty. FTT is a clinical complication often reported in toddlers and young children with severe active EoE that might require child hospitalization and the restoration of nutritional needs with large volumes of the aminoacid-based formula [12].

This study highlighted that it is fundamental to identify all delay points, starting with raising awareness among family pediatricians on EGIDs and promptly referring suspicious cases to specialized pediatric centers with a multidisciplinary team. On the other hand, allergists and gastroenterologists should promptly consider GI endoscopy with correct biopsy sampling in all those children with refractory GI symptoms, especially if complicated by atopy, peripheral eosinophilia, FTT, or feeding issues. Multidisciplinary pediatric evaluation and close collaboration with endoscopists and pathologists are pivotal in early identifying suspected cases, monitoring confirmed cases of EGIDs, and preventing potential growth complications.

Although this study first demonstrated the adverse effects of diagnostic time on growth in children with EGIDs, some limitations should be mentioned. This is a retrospective single-center study with a relatively small sample size. Moreover, these results may be influenced by the recent COVID-19 pandemic, distance from our Pediatric Hospital, or pediatric visits performed before the CPED evaluation, which we did not assess in this study. Collecting data across other pediatric centers may help reinforce these results. Further research is needed to improve EGID knowledge among pediatricians and identify non-invasive diagnostic tools and guidelines for non-esophageal forms for achieving an early diagnosis and avoiding potential complications (esophageal stenosis) and adverse effects on growth.

## Data Availability

All data generated or analyzed during this study are included in this article.

## Abbreviations

CPED: Center for pediatric eosinophilic gastrointestinal disorders
EGIDs: Eosinophilic gastrointestinal disorders
FTT: Failure to thrive
GI: Gastrointestinal
